# Determinants of puerperal sepsis among post-partum mothers in Mekelle city public hospitals, Tigray, Ethiopia, 2021: a case control study

**DOI:** 10.1186/s12905-023-02643-2

**Published:** 2023-09-21

**Authors:** Solomon Gebretsion Sahle, Solomon Weldemariam, Mihret-ab Mehari, Tomas Amare Abraha

**Affiliations:** 1 Midwifery Department, College of Health Science, Dilla University, Dilla, Ethiopia; 2 Midwifery Department, College of Health Science, Mekelle University, Mekelle, Ethiopia

**Keywords:** Determinants, Mekelle city, Puerperal sepsis, Public hospitals

## Abstract

**Background:**

Puerperal sepsis is among the leading causes of preventable maternal death not only in developing countries but also in developed countries which is usually reported as the third or fourth common direct cause of maternal death. Although the prevalence of puerperal sepsis is low, it is the significant cause of maternal mortality, morbidities and other long-term complications like secondary infertility. The aim of this study was to assess the determinants of puerperal sepsis among post-partum mothers at Mekelle city public hospitals.

**Method:**

Institution based unmatched case control study was conducted among 444 total sample size (111 cases and 333 controls) in Mekelle city public hospitals from March 21, 2021 to April 20, 2021. Consecutive sampling for the cases and systematic sampling for the controls was used. Pretested structured questionnaire was used to collect data and the data was entered into Epi data version 4.1 then cleaned, coded and edited and exported to SPSS version 23 statistical software for analysis. Logistic regression was done and variables with a *P*-value of < 0.25 on Binary logistic regression were taken to multiple logistic regression analysis. At 95% confidence interval, a *P*-value of < 0.05 was used as cut-off point to declare the association with the dependent variable.

**Results:**

Multiple logistic regression analysis revealed that rural residence (AOR: 3, 95% CI: 1.50–5.90), no ANC follow up (AOR: 2.7, 95% CI: 1.08–6.71), duration of rupture of membrane > 24 h (AOR: 4.1, 95% CI: 1.60–10.58), duration of labor > 24 h (AOR: 4.3, 95% CI: 1.86–9.92), number of vaginal examination >  = 5 (AOR: 2.8, 95% CI: 1.26–6.26), cesarean section mode of delivery (AOR: 2.8, 95% CI: 1.48–5.20) and no PNC follow up (AOR: 3.9, 95% CI: 1.60–9.36) were the determinant factors of puerperal sepsis in this study.

**Conclusion:**

The determinants of puerperal sepsis in this study were rural residence, not having antenatal care, prolonged duration of rupture of membrane, prolonged duration of labor, frequent number of vaginal examination, cesarean section and not having postnatal care. It is recommended that strengthening provision of health education on danger signs of pregnancy, parthograph utilization and avoiding of frequent vaginal examinations.

## Introduction

Puerperal sepsis (PS) is defined as a life threatening condition manifested by organ dysfunction resulting from infection during pregnancy, childbirth, post-abortion or post-partum period. There are two sets of criteria for the determination of PS: early identification of women with possible sever PS and confirmation of the diagnosis of PS through investigations [1].

PS is among the leading causes of preventable maternal death. It is not only in developing countries but also in developed countries which is usually reported as the third or fourth common direct cause of maternal death [2].

PS causes the greatest concern of all pregnancy-related infections with the prevalence range from 2.7 to 5.2 per 100,000 live births [3]. Annually 5.2 million new PS cases were occurred [4]. The prevalence of PS in South Asia falls between 3–4% of all child births, with the median prevalence of 3.84% [5]. The prevalence of PS in India, Haryana, Civil hospital Palwal is 8.68% [6] and 3.89% at tertiary health care center of Sindh, Pakistan [7].

According to world health organization (WHO) report 295,000 maternal deaths occurred during labor and child birth mainly with in the first 24 h of discharge and 15% of these were related with PS [8] and annually around 62,000 maternal death were occurred due to puerperal sepsis [4].The most significant long-term complication is secondary infertility, estimated to affect around 450,000 women each year [9].

Although maternal sepsis nowadays accounts for a small proportion of maternal deaths in high-income countries, it still causes approximately 10% of maternal deaths in Africa and Asia [10]. PS (30.9%) was the leading cause of maternal death followed by post- partum hemorrhage(21.6%)in tertiary teaching hospital Uganda [11]. It is third leading cause of death accounting 12% of maternal deaths in Nigeria [12].

The prevalence of PS in different African countries is 1.7, 0.22, 1.14 and 0.07 per 100 live births in Nigeria, Niger, Uganda and South Africa respectively [12]. The incidence of puerperal fever at Addis Ababa teaching hospitals is 8.4 [13].

In Ethiopia, PS accounted for about 13% of all maternal deaths and becomes the third leading direct cause of maternal mortality next to hemorrhage and hypertensive disorders during pregnancy [14, 15].

Global studies showed the risk factors that contribute to PS are poor hygiene practice during delivery and post-partum which is related to repeated vaginal examination of clients during labor, prolonged rupture of membrane, prolonged duration of labor, post-partum hemorrhage, retained products of conception, cesarean section delivery, endometritis, anemia, obesity, impaired immunity, advanced maternal age and poor sanitary condition and services in the health service facilities [16–19].

Despite the scientific technological developments and the advanced scientific research in various areas of knowledge, puerperal infection still constitutes a major public health problem, as evidenced by high prevalence of maternal morbidity and mortality due to maternal sepsis [20, 21].

According to the Ethiopian federal ministry of health legislation like enhancing the quality of maternal health care through expanding the number of health facilities, providing temporary maternity waiting homes for those mothers near to term pregnancy, increasing the number of health care givers especially medical doctors, midwives and health extension workers, providing training to health workers, preparing treatment guidelines and making free payments for maternal health services in the public health institutions has been made to improve maternal health [22]. The royal college of obstetricians and gynecologists (RCOG) recommended that all pregnant and recently delivered women should be informed of the signs and symptoms of genital tract infections and the prevention mechanisms. This includes antibiotic prophylaxis for preventing infections, in infection-prone conditions and obstetric procedures (prolonged rupture of membranes (PROM), meconium stained amniotic fluid, perineal tears, manual removal of placenta, operative vaginal birth and caesarean section) and the importance of personal hygiene [23].

The global initiative to improve the maternal health includes increasing the national budget allocation, increasing government funding and support for making free maternal health services, training of midwives and medical assistants, enhanced transportation systems particularly in rural areas. Interventions like preventing unplanned pregnancy through provision of family planning services, preventing or treating of pregnancy complications by having antenatal follow up skilled birth attendant and post-partum care services are other components of the initiative [24, 25].

In order to improve the maternal health service in Africa, the African leaders were also focusing on strengthening health systems through community health workers, utilizing efficient financing mechanisms. In addition to those, political partnership, addressing gender issues and educating the general population on the importance of investing in women especially in maternal health are other important areas for African governments and policy makers [24].

In Ethiopia the determinants of PS is not well described, particularly in Tigray region. To the best of our knowledge there is no tangible study on the determinants of puerperal sepsis in Tigray region. Hence this study was conducted to fill this gap by assessing the determinants of puerperal sepsis among post-partum mothers of Mekelle city in the Tigray region. It also added important variables that are not investigated in previous studies like place of delivery, anemia during labor and delivery, post-partum hemorrhage (PPH), post-natal care (PNC) follow up and pre-existed maternal health problems.

## Methods

### Study area and period

The study was conducted in Mekelle city public hospitals. Mekelle is the capital city of Tigray region, which is 783Kms far from Addis Ababa, the capital city of Ethiopia. The city administratively classified into seven sub-cities. It has total populations of 433,670 whom 219,556 and 214,114 were men and women respectively, and from those women, 52% (112,238) were found in reproductive age group that is 15–49 years [26].

In 2020, the number of women who received ANC at least one visit were 22,511 and those who received four visits and above were 10,320. The number of women attended by skilled health personnel were 17,206 and the number of still births were 382 in the city [27].

Mekelle city has one comprehensive specialized hospital, two general hospitals, one primary hospital and eleven health centers [26]. So, this study included Ayder comprehensive specialized hospital, Mekelle general hospital and Quiha general Hospital. The study period was from March 21, 2021 to April 20, 2021.

### Study design

Institution based unmatched case control study was conducted.

### Sample size determination and sampling process

Sample size was calculated using Epi Info 7.0 StatCalc program by taking assumptions of 95% confidence level, 80% power. Three controls for each case and using the percentage of controls exposed and AOR for each of the statistically significant variables in the study conducted at west Shoa zone, Oromia regional state [28] (Table 1).

**Table 1:** Sample size calculation using Epi Info 7.0 StatCalc program

**Factors**	Percent of controls exposed	AOR	Sample size for cases	Sample size for controls	Total sample size	Reference
Residence	28.6%	2.5	59	175	234	[28]
Educational status	23%	6.8	15	45	60	[28]
Total monthly income	6.1%	5.94	33	97	130	[28]
Duration of labor	18.3%	4.75	24	70	94	[28]
Mode of delivery	20.7%	3.85	30	90	120	[28]
Duration of rupture of membrane	16%	3.73	35	105	140	[28]
Number of vaginal examination	14.1%	2.3	101	303	404	[28]
Referral status during labor	27.2%	2.5	59	177	236	[28]

From the table above number of vaginal examination was the exposure variable that gives the highest sample size with total sample size of 404 (101 cases and 303 controls).So, number of vaginal examination was selected because it was the exposure variable that gives the highest sample size for cases and controls among the other variables. By adding 10% (40) contingency, the final sample size was found to be 444 (111 cases and 333 controls).

### Sampling technique and procedure

From the total public hospitals of Mekelle city, 3 hospitals were selected using simple random sampling and these public hospitals were included in the study. Using the two years report of puerperal sepsis cases in each of the selected public hospitals as a reference, proportional sample size allocation for each hospital was calculated. Cases were taken by consecutive sampling until the required sample size is achieved and three controls for each case were taken using systematic sampling. This is due to cases were rear to find out we included all cases till the required sample was reached. However, controls were easily accessible and were high in number so that we used systematic sampling method. The K^th^ interval for the selection of the controls was calculated using the two years total number of deliveries of the selected public hospitals by dividing to the total number of controls. This was every 63 post-partum mothers who had delivered in the selected public hospitals and the first was selected randomly.

### Ascertainment of cases and controls

#### Cases

Post-partum mothers diagnosed with puerperal sepsis in their charts in the selected public hospitals of Mekelle city.

#### Controls

Post-partum mothers without puerperal sepsis diagnosis in their charts in the selected public hospitals of Mekelle city.

### Data collection tool and procedures

The questionnaire was developed after in depth review of different published literatures [28–31]. The questionnaire has three parts. The first part of the questionnaire contains socio-demographic information of the participants, the second part of the questionnaire contains obstetrics and Gynecologic related information of the respondents and the third part contains maternal health related information of the study participants. Data was collected through reviewing the chart of the study participants using a structured questionnaire. The data collectors had taken training on how to collect the data.

### Data quality control

The questionnaire was designed carefully and prepared in English language. Before actual data collection time the questionnaire (tool) was discussed with the authors and pretested for validity and reliability in10% (45) of the sample size among the post-partum mothers in Wukro general hospital which is not selected for the actual data collection.

Three (3) BSc midwives were selected for the data collection. Data collectors were trained for one day on sampling techniques and data collection procedures. Data was supervised daily by the supervisors and cleaned daily by the principal investigator.

### Data processing and analysis

The data collected through the questionnaire was entered to Epi data version 4.1 then cleaned, coded, edited and exported into the SPSS version 23 for analysis. Using 95% confidence interval, the association of the independent variable with the dependent variable was done through Bivariate logistic regression, then variables with a *p*-value of < 0.25 were entered to the Multiple logistic regression. The value of *p*-value was set to < 0.25 in order to prevent missing of some important variables. Variables with a *p*- value of < 0.05 were declared as statistically significant with the dependent variable. Model goodness of fit test was checked with Hosmer–Lemeshow goodness-of-fit with the *p*-value of 0.67 and Multi collinearity was checked with variance inflation factor (VIF)/Tolerance. The value of VIF for this result ranged from 1.051 to 1.939 and the value of tolerance was ranged from 0.516 to 0.951 which indicated multi-collinearity didn’t exist among the variables. The odds ratio was calculated to identify the level of statistical significance. Finally the results were presented using text and tables.

## Results

### Socio demographic characteristics of the study participants

From the total 444 sample size, 431 charts were reviewed with the response rate of 97.1%. Thirteen charts of the study participants were not included in this study as they have missed some important variables due to inappropriate documentation. The median age of the study participants was 27 years with IQR of ± 7. Minimum age was 18 years and maximum age was 43 years with the range of 25 years. Eighty percent of the study participants were from the age group of 21–34 years.

Most, 394(91.4%), of the study participants were married. Ninety five percent of the mothers were Tegaru in ethinicity. Majority, 348(80.7%), of the study participants were Orthodox religion followers. More than two-third 309(71.7%) of the study paricipants were in urban residence. Of the study participants, 57(51.4%) cases were rural residence, whereas 65(20.3%) controls were from rural residence (Table 2).

**Table 2:** Socio demographic characteristics among post-partum mothers in mekelle city public hospitals, 2021

Variables	Cases	Controls	Total
Maternal age in years			
< 21	21(18.9%)	14(4.4%)	35(8.1%)
21–34	77(69.4%)	271(84.7%)	348(80.7%)
> = 35	13(11.7%)	35(10.9%)	48(11.1%)
Marital status			
Married	102(91.9%)	292(91.3%)	394(91.4%)
Single	5(4.5%)	11(3.4%)	16(3.7%)
Others^a^	4(3.6%)	17(5.3%)	21(4.9%)
Ethnicity			
Tigray	104(93.7%)	305(95.3%)	409(94.9%)
Afar	4(3.6%)	10(3.1%)	14(3.2%)
Amara	3(2.7%)	5(1.6%)	8(1.9%)
Religion			
Orthodox	91(82%)	257(80.3%)	348(80.7%)
Muslim	12(10.8%)	45(14.1%)	57(13.2%)
Protestant	6(5.4%)	13(4.1%)	19(4.4%)
Catholic	2(1.8%)	5(1.6%)	7(1.6%)
Residence			
Rural	57(51.4%)	65(20.3%)	122(28.3%)
Urban	54(48.6%)	255(79.7%)	309(71.7%)

Others^a^ [divorced = 13(3%), widowed = 8(1.9%)]

### Obstetrics and Gynecologic characteristics of study participants

Around two-third, 260(60.3%), of the study participants were multiparas. Among the participants, 51(45.9%) cases were primipara, but 72(22.5%) controls were primipara. Almost all, 427(99.1%), of the mothers were delivered in health institutions. Most of, 391(90.7%), were delivered at term gestational age (37–42 weeks), and 23(5.3%) were delivered at less than 37 completed weeks of gestation. Among the study participants, 10(9%), cases were post term, while 7(2.2%), controls were post term in their gestational age during their last delivery.

Eighty four percent of the study participant’s onset of labor was started spontaneously. Around three-fourth 327 (75.9%) mothers were evaluated per vaginally for less than 5 times. However, 57(51.4%), cases and 47(14.7%), controls were evaluated five or more times. Eighty one percent of the mothers were delivered within 24 h of labor duration. Among the mothers, 50(45%) cases and 31(9.7%) controls were stayed in labor for more than 24 h.

Majority, 307(71.2%), of the mothers were delivered spontaneously through SVD. However, 47(42.3%) cases and 61(19.1%) controls were delivered through cesarean section. Most, 354(82.1%), of the study participants had no episiotomy during their last labor and delivery. Of the study participants, 2(1.8%) cases and 3(0.9%) controls had IUFD birth outcome; 3(2.7%) cases and 4(1.3%) controls had still birth. Among the study participants, 30(7%), had developed PPH and 19(4.4%) were diagnosed with anemia during labor and delivery.

Around one-fifth, 80 (18.6%), mothers were referred from one to another health institution during labor. Most, 376(87.2%), of the mothers were admitted for less five days during their last labor and delivery. More than three-fourth, 335(77.7%), of the study participants had no PNC follow up after discharged from labor and delivery. However, 9 (8.1%) cases and 87 (27.2%) controls had PNC follow up of at least one visit after discharged (Table 3).

**Table 3:** Obstetrics and gynecologic characteristics among post-partum mothers in Mekelle city public hospitals, 2021

Variables	Cases	Controls	Total
Parity			
Primipara	51(45.9%)	72(22.5%)	123(28.5%)
Multipara	44(39.6%)	216(67.5%)	260(60.3%)
Grand multipara	16(14.4%)	32(10%)	48(11.1%)
ANC follow up			
No	49(44.1%)	44(13.8%)	93(21.6%)
1–3	48(43.2%)	185(57.8%)	233(54.1%)
> = 4	14(12.6%)	91(28.4%)	105(24.4%)
Obstetric complications			
Yes	27(24.3%)	64(20.0%)	91(21.1%)
No	84(75.7%)	256(80.0%)	340(78.9%)
PIH	7(25%)	16(21.3%)	23(22.3%)
APH	3(10.7%)	11(14.6%)	14(13.6%)
PROM	17(60.7%)	34(45.3%)	51(49.5%)
Oligohydramnios	0(0%)	8(10.6%)	8(7.8%)
IUGR	0(0%)	3(4.0%)	3(2.9%)
Others^a^	1(3.6%)	3(4.0%)	4(3.9%)
Place of delivery			
Home	2(1.8%)	2(0.6%)	4(0.9%)
Health institution	109(98.2%)	318(99.4%)	427(99.1%)
Duration of rupture of membrane			
< 12 h	83(74.8%)	286(89.4%)	369(85.6%)
12–24 h	10(9%)	20(6.3%)	30(7%)
> 24 h	18(16.2%)	14(4.4%)	32(7.4%)
Chorioamnionitis			
Yes	5(4.5%)	8(2.5%)	13(3%)
No	106(95.5%)	312(97.5%)	418(97%)
Duration of labor			
< = 24 h	61(55%)	289(90.3%)	350(81.2%)
> 24 h	50(45%)	31(9.7%)	81(18.8%)
Number of vaginal examination			
< 5	54(48.6%)	273(85.3%)	327(75.9%)
> = 5	57(51.4%)	47(14.7%)	104(24.1%)
Mode of delivery			
SVD	58(52.3%)	249(77.8%)	307(71.2%)
Instrumental	6(5.4%)	10(3.1%)	16(3.7%)
CS	47(42.3%)	61(19.1%)	108(25.1%)
Placental delivery			
CCT	96(86.5%)	274(85.6%)	370(85.8%)
Manual removal	15(13.5%)	46(14.4%)	61(14.2%)
PNC follow up			
No	102(91.9%)	233(72.8%)	335(77.7%)
Yes	9(8.1%)	87(27.2%)	96(22.3%)

Others^a^ Anemia = 1(0.97%), Polyhydramnios = 1(0.97%), Pyelonephritis = 1(0.97%), PTE = 1(0.97%)]

### Maternal health characteristics of the study participants

Most, 421(97.7%), of the study participants had no pre-existed medical health problems. However, 3(2.7%), cases and 7(2.2%) controls had preexisted medical health problems. Few, 10(2.3%), of mothers had STI during their last pregnancy. Among those 4(3.6%) mothers were cases and 6(1.9%) mothers were controls. Ninety six percent of the study participants had no UTI during their last pregnancy (Table 4).

**Table 4:** Maternal health characteristics among post-partum mothers in Mekelle city public hospitals, 2021

Variables	Cases	Controls	Total
Pre-existed maternal health problems			
Yes	3(2.7%)	7(2.2%)	10(2.3%)
No	108(97.3%)	313(97.8%)	421((97.7%)
Chronic hypertension			
Yes	0(0%)	3(42.9%)	3(30%)
No	3(100%)	4(57.1%)	7(70%)
Diabetic mellitus			
Yes	0(0%)	1(14.3%)	1(10%)
No	3(100%)	6(85.7%)	9(90%)
Anemia			
Yes	1(33.3%)	0(0%)	1(10%)
No	2(66.7%)	7(100%)	9(90%)
Cardiac disease			
Yes	1(33.3%)	1(14.3%)	2(20%)
No	2(66.7%)	6(85.7%)	8(80%)
HIV			
Yes	1(33.3%)	1(14.3%)	2(20%)
No	2(66.7%)	6(85.7%)	8(80%)
Others^a^	0(0%)	1(10%)	1(10%)
Yes	0(0%)	1(14.3%)	1(10%)
No	3(100%)	6(85.7%)	9(90%)
STI			
Yes	4(3.6%)	6(1.9%)	10(2.3%)
No	107(96.4%)	314(98.1%)	421(97.7%)
UTI			
Yes	6(5.4%)	10(3.1%)	16(3.7%)
No	105(94.6%)	310(96.9%)	415(96.3%)

Others^a^ [HBsAg = 1(10%)]

### Determinant factors of puerperal sepsis among post-partum mothers

In this study the independent variables that had associated with puerperal sepsis during the Binary logistic regression were age of the mother, residence, parity, ANC follow up, length of gestational age, duration of rupture of membrane, duration of labor, number of vaginal examination, mode of delivery and PNC follow up. However, Age of the mother, parity and length of gestational age were found to have no association with the dependent variable during the Multivariable analysis.

Residence was the independent variable associated with puerperal sepsis which indicates that mothers who live in the rural areas were 3 times more likely to develop puerperal sepsis than mothers who were from the urban areas [AOR:3, 95% CI 1.50–5.90]. ANC follow up was also another variable associated with puerperal sepsis. It shows that mothers who had no ANC follow up were 2.7 times more likely to have puerperal sepsis when compared to mothers who have ANC follow up of 4 or more times [AOR:2.7, 95% CI 1.08–6.71].

Duration of rupture of membrane was also found as the determinant factor of puerperal sepsis in this study. It reveals that mothers whose amniotic fluid membrane was ruptured for more than 24 h before the onset of labor were 4.1 times more likely to develop puerperal sepsis than those mothers whose amniotic fluid membrane rupture was less than 12 h before the onset of labor[AOR:4.1, CI 1.60–10.58]. Duration of labor was also the other variable strongly associated with puerperal sepsis which shows mothers who stayed in labor for more than 24 h were 4.3 times more likely to have puerperal sepsis compared to mothers who was delivered within 24 h of duration of labor during their last labor and delivery [AOR:4.3, 95% CI 1.86–9.92].

Furthermore, the number of vaginal examination during labor and delivery was another independent variable associated with puerperal sepsis that indicates mothers who undergone vaginal examination of five or more times were 2.8 times more likely to develop puerperal sepsis than mothers who had vaginal examination of less than five times during their last labor and delivery [AOR: 2.8, 95% CI 1.26–6.26]. In addition to this mode of delivery was also identified as the determinant factor of puerperal sepsis. It shows that mothers who delivered through cesarean section were 2.8 times more likely to develop puerperal sepsis when compared to those who delivered by SVD [AOR:2.8, 95% CI 1.48–5.20].

Moreover, PNC follow up was also another variable associated with puerperal sepsis which indicated mothers who had no PNC follow up were 3.9 times more likely to develop puerperal sepsis than those who have PNC follow up of at least one visit after discharged [AOR:3.9, 95% CI 1.60–9.36**]** (Table 5).

**Table 5:** Multiple logistic regression analysis showing determinants of puerperal sepsis among post-partum mothers 2021

Variable	Category	Puerperal sepsis		COR (95% CI)	AOR (95% CI)
		Cases = 111	Controls = 320		
**Age in years**	< 21	21(18.9%)	14(4.4%)	5.3 (2.56–10.87)	2.4 (0.81–7.30)
	21–34	77(69.4%)	271(84.7%)	1	1
	> = 35	13(11.7)	35(10.9%)	1.3(0.66–2.60)	0.5 (0.14–1.82)
**Residence**	Rural	57(51.4%)	65(20.3%)	4.1 (2.61–6.57)	3 (1.50–5.90)^**^
	Urban	54(48.6%)	255(79.7%)	1	1
**Parity**	Primipara	51(45.9%)	72(22.5%)	3.5 (2.14–5.64)	2 (0.97–4.12)
	Multipara	44(39.6%)	216(67.5%)	1	1
	G.multipara	16(14.4%)	32(10.0%)	2.5 (1.24–4.85)	0.9 (0.28–3.45)
**ANC follow up**	No	49(44.1%)	44(13.8%)	7.2 (3.61–14.50)	2.7 (1.08–6.71)^*^
	1–3	48(43.2%)	185(57.8%)	1.7 (0.88–3.22)	0.9 (0.45–2.15)
	> = 4	14(12.6%)	91(28.4%)	1	1
**Gestational age**	< 37 weeks	9(8.1%)	14(4.4%)	2.1 (0.88–4.98)	1.7 (0.51–5.49)
	37–42 weeks	92(82.9%)	299(93.4%)	1	1
	> 42 weeks	10(9.0%)	7(2.2%)	4.6 (1.72–12.54)	3.4 (0.95–12.28)
**Duration of rupture of membrane**	> 24 h	18(16.2%)	14(4.4%)	4.4 (2.11–9.29)	4.1 (1.60–10.58)^**^
	12–24 h	10(9.0%)	20(6.3%)	1.7 (0.78–3.83)	2.4 (0.90–6.40)
	< 12 h	83(74.8%)	286(89.4%)	1	1
**Duration of labor**	> 24 h	50(45.0%)	31(9.7%)	7.6 (4.51–12.93)	4.3 (1.86–9.92)^**^
	< = 24 h	61(55.0%)	289(90.3%)	1	1
**Vaginal examination**	> = 5	57(51.4%)	47(14.7%)	6.1 (3.78–9.95)	2.8 (1.26–6.26)^*^
	< 5	54(48.6%)	273(85.3%)	1	1
**Mode of delivery**	CS	47(42.3%)	61(19.1%)	3.3 (2.05–5.32)	2.8 (1.48–5.20)^**^
	Instrumental	6(5.4%)	10(3.1%)	2.6 (0.90–7.37)	2.5 (0.60–10.10)
	SVD	58(52.3%)	249(77.8%)	1	1
**PNC follow up**	No	102(91.9%)	233(72.8%)	4.2 (2.05–8.73)	3.9 (1.60–9.36)^**^
	Yes	9(8.1%)	87(27.2%)	1	1

^*^ = *p*-value < 0.05 and

^**^ = *p*-value < 0.01

## Discussion

This study was conducted to identify the determinant factors of puerperal sepsis among post-partum mothers in Mekelle city public hospitals. Hence, the independent variable that had association with puerperal sepsis during the Multiple logistic regression analysis were residence, ANC follow up, duration of rupture of membrane, duration of labor, number of vaginal examination, mode of delivery and PNC follow up.

Mothers from the rural residence were 3 times more likely to develop puerperal sepsis compared to those who were from urban areas. This is in line with the study conducted in west shoa zone, Oromia regional state [28]. This may be related to poor sanitation and hygienic practice, educational status, low awareness on health seeking behavior and non-availability of health facility at nearby. So that rural dwellers were more likely to die due to puerperal sepsis than whose residence is urban. This is supported by the study conducted in Uganda in which puerperal sepsis was the leading direct cause of maternal death [11].

Mothers who have no ANC follow up were 2.7 times more likely to develop puerperal sepsis than those who have ANC follow up of 4 or more times. This is supported by the study conducted in Pakistan [32]. This might be due to mothers who have ANC follow up will have information on health promotion, disease prevention with supplementation and diversified nutrition and easily identification of danger signs during pregnancy. They are also more likely to give birth at health facility with sterile equipment’s by skilled birth attendant. However, mothers who had no ANC are at increased risk to have maternal morbidity and mortality. This is supported by the study conducted in Uganda in which mothers who had no ANC are 3.6 more likely to die [11].

This study also reveals that mothers who had prolonged rupture of membrane for more than 24 h before the onset of labor and delivery were 4.1 more likely to develop puerperal sepsis compared to those whose amniotic fluid membrane rupture was less than 12 h before the onset of labor. This is consistent with the study conducted in west shoa, Oromia regional state [28].It is also supported by the study conducted in India [6]. This is due to the prolonged opening of the cervix which leads the microorganisms to ascend to the uterus and adjacent reproductive organs.

This study also shows that mothers who stayed in labor for more than 24 h were 4.3 times more likely to develop puerperal sepsis than those who delivered within 24 h of labor duration. This is supported by the study conducted in Pumwani maternity hospital, Nairobi, Kenya [30]. It is also in line with the study done at west shoa, Oromia regional state [28]. This is because of prolonged labor attracts several vaginal examinations. Prolonged state of an open cervix, with rupture of membrane often leads to ascend microorganisms from the vagina that will cause puerperal sepsis.

This study reveals that mothers who had vaginal examination of five or more times were 2.8 times to develop puerperal compared to those who had less than five of vaginal examination. This is in line with the study conducted in west shoa, Oromia regional state [28]. It is also consistent with the study done at Pakistan, India and Kenya, [32, 30, 6]. This is might be due to frequent vaginal examination which helps the pathogenic organisms to pass through the open cervix that will cause puerperal sepsis.

This study found that mode of delivery was significantly associated with puerperal sepsis that shows mothers who delivered by CS were 2.8 times more likely to develop puerperal sepsis compared to those who delivered by SVD. This is in line with the study done in Kenya [30] and India [6]. It is also supported by the study done in west shoa, Oromia regional state [28]. This may be related to opening of internal organs with poor sterilization techniques and no provision or late administration of prophylactic antibiotics. Single dose pre-operative anti-microbial provision for all women undergoing cesarean delivery is recommended. This practice decreases the risk of developing puerperal sepsis by 65 to 75% [33].

However, it is inconsistent with the study done in university of Gondar referral hospital that shows mothers who delivered by cesarean section were 2 times less likely to have puerperal sepsis than those who delivered through SVD [34]. This may be due to the difference in study setting. Sterility techniques, pre-operative and post-operative cares given in this study area might be poor comparing to Gondar referral hospital.

This result found that mothers who had no PNC follow up were 3.9 times more likely to develop puerperal sepsis when compared to those who had PNC follow up of at least one visit after they are discharged. This might be related to hygiene, nutrition and early detection of post-partum danger signs with the information obtained through counseling.

### Limitations

This study has used secondary data by reviewing the chart of the study participants. Due to this, some important variables like socio economic and educational status were missed. In addition to this, it was done at public hospitals only and was conducted retrospectively.

## Conclusion

The determinant factors of puerperal sepsis were rural residence, not having antenatal care, prolonged duration of rupture of membrane, prolonged duration of labor, frequent number of vaginal examination, cesarean section mode of delivery and not having postnatal care. Most of these factors are related to the care given during pregnancy, labor and delivery and post-partum period. Strengthening provision of health education and providing quality health care to mothers during ANC, delivery and PNC follow up as well as proper utilization of parthograph is recommended.

## Data Availability

The datasets generated and/or analyzed during the current study are available from the corresponding author on reasonable request.

## Abbreviations

APH: Antepartum hemorrhage
AOR: Adjusted Odds Ratio
ANC: Ante Natal Care
CI: Confidence Interval
COR: Crude odds ratio
C/S: Cesarean Section
HbsAg: Hepatitis B antigen
HIV: Human immunodeficiency virus
IQR: Interquartile range
IUFD: Intra Uterine Fetal Death
MRN: Medical Registration Number
OR: Odds Ratio
PIH: Pregnancy induced hypertension
PNC: Post-natal care
PPH: Post-Partum Hemorrhage
PROM: Prolonged Rupture of Membrane
PTE: Pulmonary thromboembolism
PS: Puerperal Sepsis
STI: Sexually transmitted infections
SVD: Spontaneous Vertex Delivery
UTI: Urinary tract infection
VIF: Variance inflation factor
WHO: World Health Organization
